# Tinnitus Is Associated With Improved Cognitive Performance in Non-hispanic Elderly With Hearing Loss

**DOI:** 10.3389/fnins.2021.735950

**Published:** 2021-10-28

**Authors:** Yasmeen Hamza, Fan-Gang Zeng

**Affiliations:** Center for Hearing Research, Department of Anatomy and Neurobiology, Biomedical Engineering, Cognitive Sciences, Otolaryngology–Head and Neck Surgery, University of California, Irvine, Irvine, CA, United States

**Keywords:** tinnitus, cognition, elderly, Hispanic, hearing loss

## Abstract

Because hearing loss is a high-risk factor for cognitive decline, tinnitus, a comorbid condition of hearing loss, is often presumed to impair cognition. The present cross-sectional study aimed to delineate the interaction of tinnitus and cognition in the elderly with and without hearing loss after adjusting for covariates in race, age, sex, education, pure tone average, hearing aids, and physical well-being. Participants included 643 adults (60–69 years old; 51.3% females) from the National Health and Nutrition Examination Survey (NHANES, 2011–2012), and 1,716 (60–69 years old; 60.4% females) from the Hispanic Community Health Study (HCHS, 2008–2011). Multivariable linear and binary logistic regression was used to assess the association between tinnitus and cognition in the two sub-cohorts of normal hearing (NHANES, *n* = 508; HCHS, *n* = 1264) and hearing loss (NHANES, *n* = 135; HCHS, *n* = 453). Cognitive performance was measured as a composite z-score from four cognitive tests: The Consortium to Establish a Registry for Alzheimer’s Disease (CERAD)-word learning, CERAD-animal fluency, CERAD-word list recall, and the digit symbol substitution test (DSST) in NHANES, and a comparable Hispanic version of these four tests in HCHS. Multivariable linear regression revealed no association between tinnitus and cognition, except for the NHANES (non-Hispanic) participants with hearing loss, where the presence of tinnitus was associated with improved cognitive performance (Mean = 0.3; 95% CI, 0.1–0.5; *p*, 0.018). Using the 25th percentile score of the control (i.e., normal hearing and no tinnitus) as a threshold for poor cognitive performance, the absence of tinnitus increased the risk for poor cognitive performance (OR = 5.6, 95% CI, 1.9–17.2; *p*, 0.002). Sensitivity analysis found a positive correlation between tinnitus duration and cognitive performance in the NHANES cohort [*F*(4,140), 2.6; *p*, 0.037]. The present study finds no evidence for the assumption that tinnitus impairs cognitive performance in the elderly. On the contrary, tinnitus is associated with improved cognitive performance in the non-Hispanic elderly with hearing loss. The present result suggests that race be considered as an important and relevant factor in the experimental design of tinnitus research. Future longitudinal and imaging studies are needed to validate the present findings and understand their mechanisms.

## Introduction

Hearing loss is one of the most prevalent conditions in the elderly, including nearly half of those aged 65–84 years (Nash et al., 2011; Davis et al., 2016). Not only does hearing loss contribute to age-related cognitive decline (Lin, 2011; Lin et al., 2011a,b; Deal et al., 2017), but also is a leading modifiable risk factor that may prevent or delay 40% of dementia cases (Livingston et al., 2020). Tinnitus, or ringing in the ear, affects 15% of the general population (Nondahl et al., 2010; McCormack et al., 2016). Tinnitus is not only a common comorbid condition of hearing loss in the elderly, with about 80% overlap (Lockwood et al., 2002; Baguley et al., 2013) but also a concomitant symptom of dementia, with 52% overlap (Spiegel et al., 2018).

Because of this high comorbidity, tinnitus has been often assumed to impair cognition (Hallam et al., 2004; Savastano, 2008; Tegg-Quinn et al., 2016; Chu et al., 2020; Lee, 2020). For example, Tegg-Quinn et al. (2016) reviewed 18 relevant studies to show an association between tinnitus and some aspects of cognitive function, such as executive control of attention. In a meta-analysis study involving 38 records from 1,863 participants, Clarke et al. (2020) found that tinnitus is additionally associated with lower processing speed and poorer short-term memory. Based on a national population retrospective study, Chu et al. (2020) showed that tinnitus is an independent risk factor for subsequent Alzheimer’s and Parkinson’s disease, suggesting a role of tinnitus in age-related cognitive decline.

There are three knowledge gaps about the assumption that tinnitus impairs cognition in these previous studies. First, most studies did not control for potential interactive factors such as age, sex, race, hearing loss, education, anxiety, depression, and physical wellbeing, potentially confounding the role of tinnitus in cognition (Mohamad et al., 2016; Tegg-Quinn et al., 2016; Jafari et al., 2019). Second, if we accepted the aforementioned tinnitus-impairing cognition assumption, it remains unclear whether tinnitus inserts a general influence on the global cognitive function or affects specific domains of cognition such as attention, executive function, episodic or working memory (Albert et al., 2011; Veríssimo et al., 2021). Third, tinnitus is highly heterogeneous, with different tinnitus attributes and types potentially affecting different domains of cognitive function (Baguley et al., 2013).

Here we partially addressed these three knowledge gaps by studying the relation of tinnitus to cognition in a specific age group of elderly adults (60–69 years old) who participated in the National Health and Nutrition Examination Survey 2011–2012 (NHANES) and the Hispanic Community Health Study 2008–2011 (HCHS). First, we delineated the tinnitus role in cognition, among different hearing statuses, after controlling for age, sex, race, education, and physical wellbeing covariates. Second, we examined not only a global cognitive measure but also several separate cognitive domains. Third, in conditions where tinnitus affects cognition, we examined the relationship between specific tinnitus attributes and the affected cognitive function. Our overarching hypothesis was that tinnitus impairs cognitive function even if hearing loss and other covariates are accounted for.

## Materials and Methods

### Participants and Cohorts

The current cross-sectional study includes cohorts from the NHANES and the HCHS.

**FIGURE 1:**
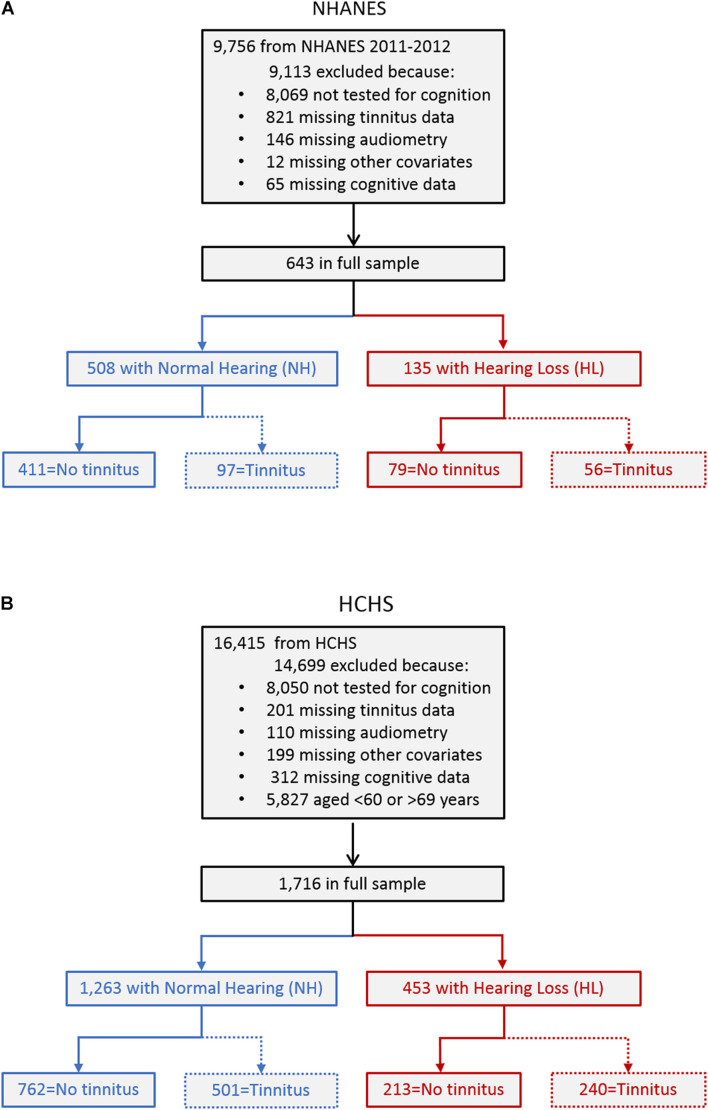
Experimental design flowchart in the NHANES [panel **(A)**] and HCHS **(B)** database.

The NHANES is a biannual United States-representative cross-sectional study. The 2011–2012 cycle entailing 9,756 individuals was chosen, as it was the only cycle that assessed more than one cognitive domain. Figure 1A shows the process of identifying the participants for the present study. A total of 9,113 participants were excluded, including 8,069 who did not participate in the cognitive tests because, within that cycle, cognitive tests were only administered to participants aged between 60 and 69 years, and 1,044 who had missing data in various tests. The resulting full sample included 643 participants who had completed an assessment on tinnitus, pure-tone audiometry, other covariates, and cognitive tests. To delineate the role of hearing loss in tinnitus effect on cognition, the full sample was divided into two sub-cohorts: 508 participants with normal hearing (blue left box) and 135 with hearing loss (red right box). Hearing loss was defined as a threshold greater than 25 dB HL based on the unaided better-ear pure-tone average (PTA) of 0.5, 1, 2, and 4 kHz [World Health Organization (WHO), 2008], as in previous studies assessing the association between hearing loss and cognition in the elderly (Lin, 2011; Lin et al., 2011a,b; Deal et al., 2015, 2017). Finally, to examine the interactive effects of hearing loss and tinnitus on cognition, the normal hearing and hearing loss sub-cohorts were divided into four groups with no tinnitus and tinnitus, respectively (the bottom row).

To confirm NHANES findings or to assess their generalizability to the Hispanic population, the HCHS cohort was often chosen (Cruickshanks et al., 2015; Golub et al., 2017, 2020). The HCHS is a multicenter United States community-based prospective study in Hispanic/Latino populations conducted between 2008 and 2011 entailing 16,415 individuals. Figure 1B shows the process of identifying the participants for the present study. A total of 14,699 participants were excluded, including 8,050 who did not participate in the cognitive tests, and 822 who had missing data in various tests. An additional 5,827 participants were excluded to match the NHANES cohort age range of 60–69 years. The resulting full sample included 1,716 participants who had completed an assessment on tinnitus, pure-tone audiometry, other covariates, and cognitive tests. The full sample was divided into two sub-cohorts: 1,263 participants with normal hearing (blue left box) and 453 with hearing loss (red right box). The same definitions in NHANES regarding hearing loss and tinnitus were used to divide the cohort into four groups (the bottom row).

### Cognitive Performance

Cognitive performance was the primary outcome measure. Four tests were conducted to assess cognitive function. The results from the four cognitive tests were normalized by standard deviations from the full-sample mean, then averaged to yield a single cognitive z-score. For example, in the NHANES cohort, a z-score of zero is equivalent to the mean of the 643 participants, and a z-score of 1.0 indicates a value that is one standard deviation above the mean performance. A single cognitive score provides a better measure of global cognitive function than individual scores for clinical evaluation of patients with cognitive impairment, especially when individual test scores do not show concordance (Mistridis et al., 2015; Palumbo et al., 2020).

The four tests administered are as follows: To test immediate learning memory, the Consortium to Establish a Registry for Alzheimer’s Disease (CERAD)-word learning test (Morris et al., 1989) in NHANES, and its HCHS-equivalent: the Spanish-English Verbal Learning Test (SEVLT: González et al., 2001) was used, in which the participant was presented with unrelated common words randomly in three trials and recalled them immediately (10 in CERAD and 15 in SEVLT). To test delayed learning memory, the CERAD-word list recall, and SEVLT-recall were used, in which the participant was asked to recall as many words as possible from the words learned in the first test after a short delay (CERAD) or a distracting word list (SEVLT). To test executive function, the CERAD-Animal Fluency test was used in NHANES, in which the participant named as many animals as possible in 1 min, and the Word Fluency Test (Spreen and Strauss, 1998) was used in HCHS, in which the participant named as many words as possible that begin with certain letters in two trials of 1 min each. To test sustained attention, processing speed, and working memory, the Digit Symbol Substitution Test (Wechsler, 1997; Lezak et al., 2004) was used in both NHANES and HCHS. The participant was shown a key containing nine numbers and their paired symbols, then asked to, in 2 min in NHANES and 90 s in HCHS, transcribe as many symbols as possible corresponding to the numbers in a contiguous box that contains up to 133 symbols. High correlation was present between the individual tests in NHANES (*r*, 0.4–0.7; *p* < 0.001) and HCHS (*r*, 0.3–0.7; *p* < 0.001).

### Tinnitus

Tinnitus was the primary exposure under test. It was encoded as a categorical variable, defined as whether the participant had experienced “Tinnitus” or “No Tinnitus” in the past year before examination. For those with tinnitus, three tinnitus-specific attributes were further examined in the NHANES cohort. Tinnitus duration was encoded as a five-level categorical variable, including less than 3 months, 3 months to a year, 1 to 4 years, 5 to 9 years, and ten or more years. Tinnitus severity was encoded as a five-level categorical variable, including no problem, a small problem, a moderate problem, a big problem, a very big problem. Tinnitus frequency was encoded as a five-level categorical variable, including less frequently than once a month, at least once a month, at least once a week, at least once a day, almost always. The reference category was set to the category of least magnitude. For example, for the tinnitus duration attribute, the shortest duration (less than 3 months) served as a reference, namely coded as 0, whereas the longest duration (ten or more years) was coded as 4. For the HCHS cohort, only data on tinnitus frequency was available and was encoded similar to the NHANES.

### Covariates

Covariates included age, sex, education, physical well-being score, PTA in both NHANES and HCHS cohorts, and additionally race in the NHANES cohort. Hearing aid use was added as a covariate only in the hearing loss sub-cohorts of both the NHANES and HCHS. The selection of covariates was based on a statistically significant association with cognitive performance using univariable linear regression models in NHANES (see Table 1). Age was a continuous variable, ranging from 60 to 69 years old. Sex was a binary variable as either female or male. Education was coded as a four-level categorical variable, including less than ninth grade, high school, high school graduate or some college, and college degree or higher. The physical well-being score was a continuous variable, ranging from zero to five based on the presence of the following risk factors: coronary artery disease, hypertension, history of transient ischemic attack, impaired glucose tolerance, and diabetes (Golub et al., 2020). The PTA was a continuous variable, ranging from −7.5 to 100 dB HL. The race was coded as a binary variable as either Hispanic or non-Hispanic in the NHANES cohort. Additionally, in the hearing loss sub-cohorts of both the NHANES and HCHS, hearing aid use was coded as a binary variable as either worn hearing aids previously or not.

**TABLE 1:** Covariate’s association with cognition using univariable linear regression.

**Covariate**	**Cognitive Z-score difference (95% CI)**	***p***
Age	−0.03 (−0.1–0.0)	0.004*
Gender	0.4 (0.3–0.5)	< 0.001*
**Educational level**		
Less than ninth grade	Reference	Reference
High school (HS)	0.6 (0.4–0.8)	< 0.001*
HS graduate- some college	1.1 (0.9–1.3)	< 0.001*
College degree or higher	1.4 (1.2–1.6)	< 0.001*
Physical well-being score	−0.1 (−0.2 to −0.1)	< 0.001*
Psychological well-being score^a^	0.0 (0.02–0.01)	0.25
PTA	−0.01 (−0.02 to −0.01)	< 0.001*
Hearing aid	0.2 (−0.1−0.5)	0.22
Hearing aid in hearing loss	0.5 (0.1–0.8)	0.007*
Race	0.4 (0.2–0.5)	< 0.001*

^*a*^The psychological well-being score is a depression screener score obtained by the patient health questionnaire (PHQ; Spitzer et al., 1999), that assesses the frequency of symptoms of depression over the past 2 weeks using nine items. * indicates a significant association between the covariate and cognition (*p* < 0.05).

### Statistical Analysis

**FIGURE 2:**
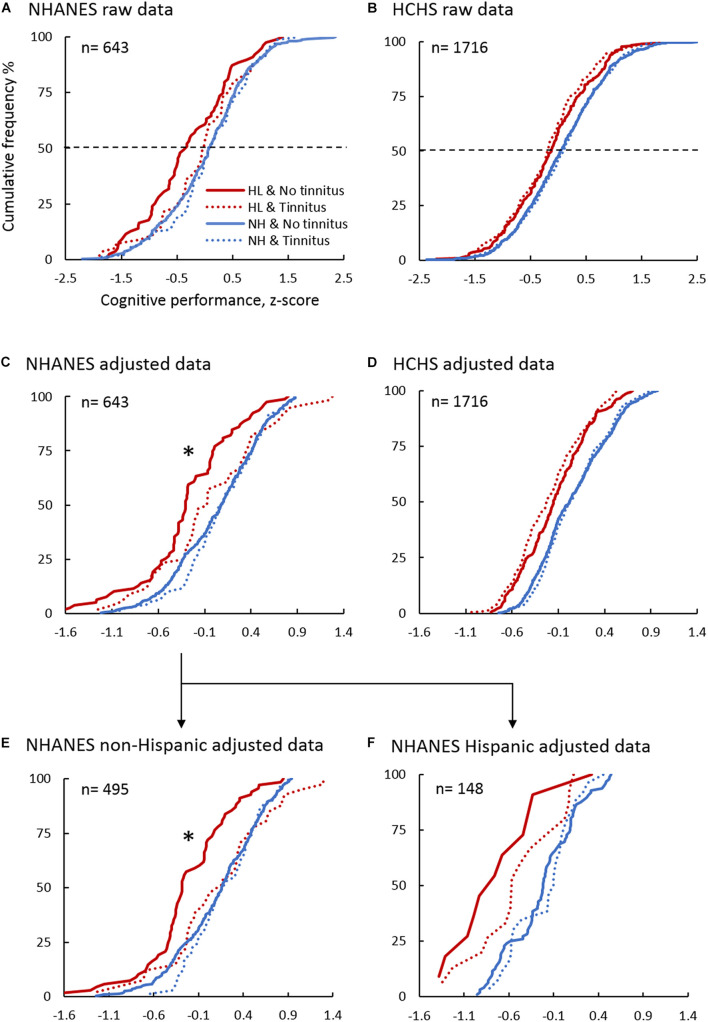
Cumulative Frequency Distribution of the Cognitive Performance Z-score in the NHANES and HCHS Sub-cohorts. Panels show the raw data in NHANES **(A)** and HCHS **(B)**, the adjusted cognitive performance using multivariable linear regression in NHANES **(C)**, HCHS **(D)**, NHANES non-Hispanic **(E)**, and NHANES Hispanic **(F)** sub-cohorts. Blue lines denote normal hearing, and red lines are of hearing loss. Solid lines are of No tinnitus, and dashed lines are of tinnitus. ^∗^indicates a significant association between tinnitus and improved cognition in the hearing loss sub-cohort (*p* < 0.05).

Analysis was done for the NHANES and HCHS cohorts separately. First, descriptive analysis was performed on all variables, with continuous variables being described in terms of means (SDs) and range, while categorical variables were in frequencies and proportions (Tables 2, 3). Raw cumulative frequency curves were generated as a function of cognitive z-score for the four groups to visualize the overall trends in the data (Figures 2A,B).

**TABLE 2:** Participant characteristics and test scores of the NHANES sub-cohorts.

	**NHANES sub-cohorts**			
**Variable**	**Normal hearing (508)**		**Hearing loss (135)**	
	**No tinnitus (411)**	**Tinnitus (97)**	**No tinnitus (79)**	**Tinnitus (56)**
Age, mean (SD) (range), y	63.7 (2.7) (60–69)	63.7 (2.7) (60–69)	64.2 (2.7) (60–69)	64.5 (3.2) (60–69)
**Gender**				
Females, No. (%)	202 (49.1)	34 (35.1)	56 (70.9)	38 (67.9)
Males, No. (%)	209 (50.9)	63 (64.9)	23 (29.1)	18 (32.1)
**Educational level, No. (%)**				
Less than ninth grade	37 (9.0)	8 (8.2)	11 (13.9)	8 (14.3)
High school (HS)	142 (34.5)	35 (36.1)	40 (50.6)	19 (33.9)
HS graduate- some college	119 (29.0)	33 (34.0)	20 (25.3)	23 (41.1)
College degree or higher	113 (27.5)	21 (21.7)	8 (10.1)	6 (10.7)
Physical well-being score, mean (SD) (range)	1.1 (1.1) (0–4)	1.4 (1.2) (0–5)	1.1 (1.0) (0–4)	1.3 (1.3) (0–4)
PTA^a^, mean (SD) (range), dB HL	13.6 (6.0) (−7.5–25)	14.9 (6.3) (1.3–25)	36.0 (10.1) (26.3–70)	35.6 (8.4) (26.3–60)
Hearing aids, No. (%)	N/A	N/A	12 (15.2)	12 (21.4)
DSST score, mean (SD) (range), z-score	0.0 (1.0) (−2.8–2.8)	0.2 (0.9) (−1.8–2.3)	−0.2 (0.9) (−2.3–1.7)	−0.1 (1.0) (−2.6–2.4)
CERAD learning score, mean (SD) (range), z-score	0.0 (1.0) (−4.6–2.3)	0.1 (0.9) (−2.2–2.1)	−0.4 (1.1) (−3.2–1.6)	−0.1 (0.9) (−2.4–2.1)
CERAD recall score, mean (SD) (range), z-score	0.0 (1.0) (−3.0–1.9)	0.1 (0.9) (−2.0–1.9)	−0.3 (1.0) (−3.0–1.4)	−0.1 (1.0) (−3.0–1.9)
Animal fluency Test score, mean (SD) (range), z-score	0.0 (1.0) (−2.6–4.0)	0.0 (1.0) (−1.7–3.1)	−0.2 (0.9) (−2.4–2.9)	0.1(1.0) (−2.1–2.4)
Cognitive performance, mean (SD) (range), z-score	0.0 (0.8) (−2.2–2.4)	0.1 (0.7) (−1.9–1.7)	−0.3 (0.8) (−1.7–1.4)	0.0 (0.8) (−1.9–2.0)

*^a^Pure-Tone Average threshold of four frequencies (0.5, 1, 2, and 4 KHz) in the better hearing ear.*

**TABLE 3:** Participant characteristics and test scores of the HCHS sub-cohorts.

	**HCHS sub-cohorts**			
**Variable**	**Normal hearing (1263)**		**Hearing loss (453)**	
	**No tinnitus (762)**	**Tinnitus (501)**	**No Tinnitus (213)**	**Tinnitus (240)**
Age, mean (SD) (range), y	63.4 (2.7) (60–69)	63.3 (2.7) (60–69)	63.8 (2.8) (60–69)	64.2 (3.0) (60–69)
**Gender**				
Females, No. (%)	468 (61.4)	364 (72.7)	84 (39.4)	120 (50.0)
Males, No. (%)	294 (38.6)	137 (27.3)	129 (60.6)	120 (50.0)
**Educational level, No. (%)**				
Less than ninth grade	262 (34.4)	191 (38.1)	86 (40.4)	106 (44.2)
High school (HS)	210 (27.6)	139 (27.7)	72 (33.8)	83 (34.6)
HS graduate- some college	177 (23.2)	123 (24.6)	38 (17.8)	47 (19.6)
College degree or higher	113 (14.8)	48 (9.6)	17 (8.0)	4 (1.7)
Physical well-being score, mean (SD) (range)	1.7 (1.2) (0–5)	1.7 (1.2) (0–5)	1.8 (1.1) (0–5)	2.0 (1.1) (0–4)
PTA^a^, mean (SD) (range), dB HL	15.5 (5.4) (0–25)	16.3 (5.4) (0–25)	34.1 (9.4) (26.3–83.8)	37.3 (12.7) (26.3–100)
Hearing aids, No. (%)	N/A	N/A	11 (5.2)	20 (8.4)
DSST score, mean (SD) (range), z-score	0.1 (1.0) (−2.4–3.4)	0.0 (1.0) (−2.4–3.2)	−0.1 (1.0) (−2.1–2.9)	−0.2 (0.9) (−2.3–2.9)
CERAD learning score, mean (SD) (range), z-score	0.1 (1.0) (−2.9–3.3)	0.1 (1.0) (−2.7–2.9)	−0.2 (1.0) (−2.5–2.5)	−0.2 (1.0) (−3.5–2.7)
CERAD recall score, mean (SD) (range), z-score	0.1 (0.9) (−2.7–2.6)	0.1 (1.0) (−2.7–2.3)	−0.1 (1.0) (−2.7–2.3)	−0.3 (1.0) (−2.7–2.3)
Animal fluency Test score, mean (SD) (range), z-score	0.1 (1.1) (−2.6–4.4)	0.0 (1.0) (−2.4–3.2)	−0.1 (1.0) (−2.3–2.7)	−0.1(0.9) (−2.4–2.7)
Cognitive performance, mean (SD) (range), z-score	0.1 (0.8) (−2.4–2.5)	0.1 (0.7) (−2.1–1.9)	−0.1 (0.7) (−2.2–2.0)	−0.2 (0.7) (−2.1–2.3)

*^*a*^Pure-tone average threshold of four frequencies (0.5, 1, 2, and 4 KHz) in the better hearing ear.*

Second, to account for the significant covariate effect on cognition, multivariable linear regression models were used to obtain adjusted cognitive z-score, and to assess the association between tinnitus and cognition in the two sub-cohorts (normal hearing and hearing loss):


Adjusted⁢cognitive⁢z-score=β⁢0+β⁢1*Age+β⁢2*Sex+β⁢3*Education+β⁢4*Physical⁢Well-being+β⁢5*PTA+β⁢6*Race+β⁢7*Hearing⁢Aid+β⁢8*Tinnitus+ε

The analysis utilized the cognitive z-score for global cognition and was replicated for each of the individual cognitive test z-scores. Regression β*8* coefficients and 95% confidence intervals (CI) were reported, which is the difference in adjusted z-score based on tinnitus status. Note that race was used only in the NHANES cohort, and hearing aid was used only in the hearing loss sub-cohort. Cumulative frequency curves were generated as a function of the adjusted cognitive z-score (Figures 2C,D).

Third, to test the consistency in the results between the NHANES and HCHS cohorts, the NHANES cohort was stratified into Hispanic and Non-Hispanic groups. Multivariable linear regression was conducted in the two groups to derive adjusted cognitive z-score and to assess the association between tinnitus and cognition. Cumulative frequency curves were generated as a function of the adjusted cognitive z-score (Figures 2E,F).

**FIGURE 3:**
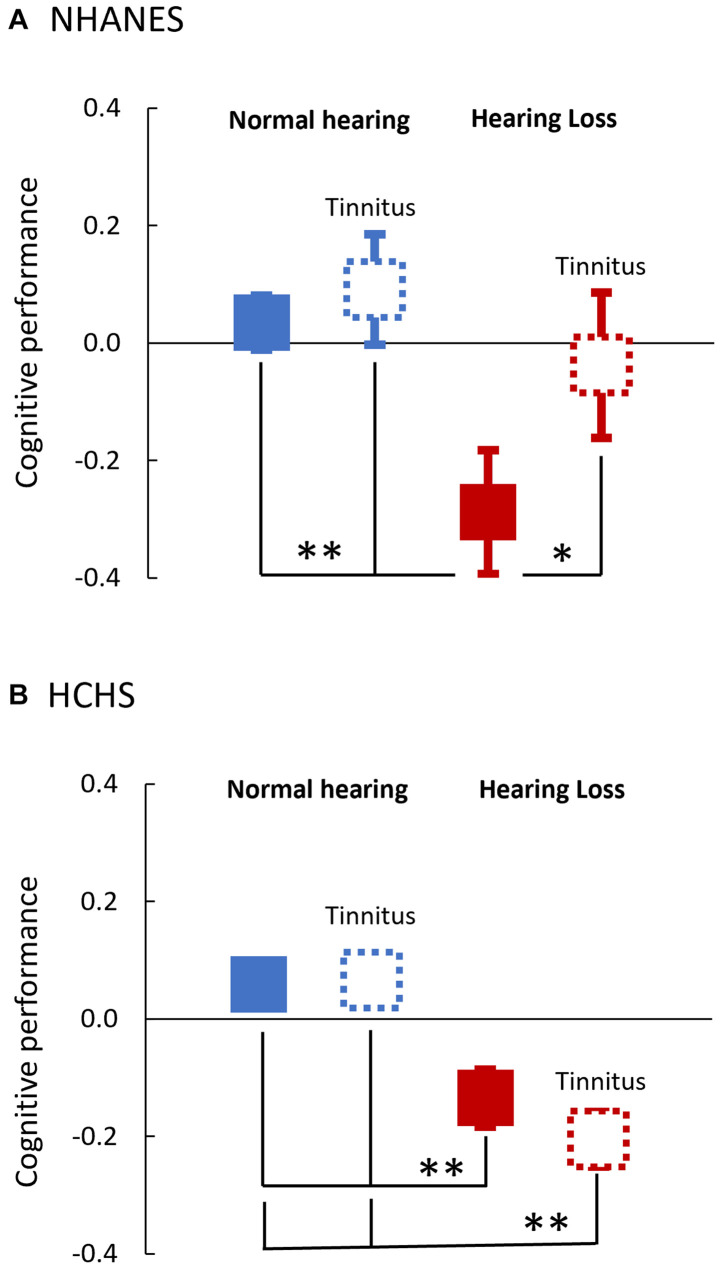
Adjusted Mean Cognitive Performance of the Four Study Groups in both NHANES **(A)** and HCHS **(B)**. One-way ANOVAs with the *post hoc* Bonferroni test were used for between-groups comparison of the adjusted cognitive z-score. Boxes denote the adjusted mean and error bars denote the 95 CI%. Blue denotes normal hearing and red denotes hearing loss. Solid lines are of No tinnitus, and dashed lines are of tinnitus. *, **indicates a significant difference in the adjusted cognitive performance between groups (^∗^*p* < 0.05; ^∗∗^*p* < 0.001).

Fourth, to examine the between-group effect on cognitive performance, one-way ANOVAs were performed with the independent variable “Study Groups (4)” and dependent variable “adjusted z-score.” *Post hoc* tests with Bonferroni corrections for multiple comparisons were conducted for pair-wise comparison between the four groups (Figure 3).

**FIGURE 4:**
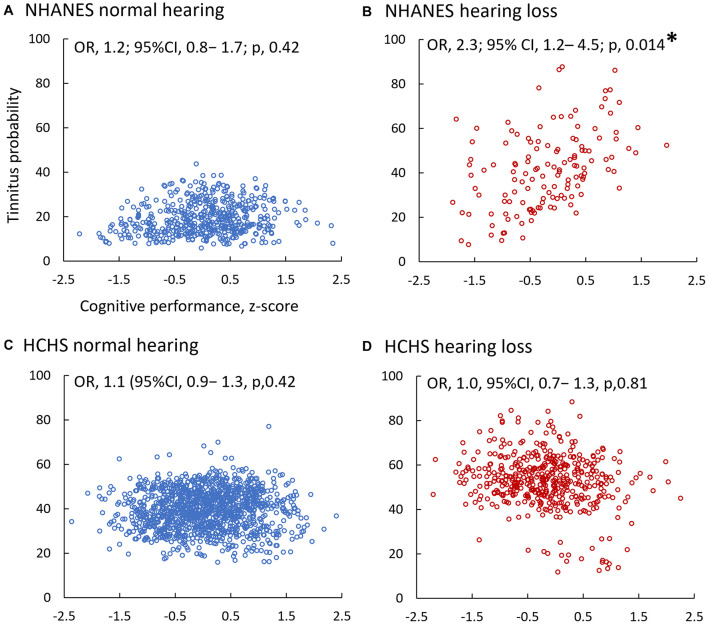
Adjusted Tinnitus Probability as a Function of Cognitive Performance in the NHANES **(A,B)** and HCHS **(C,D)** Sub-Cohorts. Multivariable binary logistic regression models predicted the adjusted probability. Blue circles denote normal hearing **(A,C)**, and red circles are of hearing loss **(B,D)**. ^∗^indicates a significant association between tinnitus and cognition (*p* < 0.05).


Fifth, to predict tinnitus probability based on cognitive performance, a multivariable binary logistic regression model was used with tinnitus being the binary outcome and cognitive z-score being the predictor under test (Figure 4):

Probability⁢(Tinnitus)=

$$\frac{1}{1+e^{-(\beta 0+\beta 1*Age+\beta 2*Sex+\beta 3*Education+\beta 4*}}$$

$${}^{P\,h\,y\,s\,i\,c\,a\,l\,W\,e\,l\,l-b\,e\,i\,n\,g+\beta \,5*\text{PTA}+\beta \,6*\text{Race}+\beta \,7*\mathrm{Hearing}\,\mathrm{Aid}}$$

$${}^{+\beta 8*Cognitiveperformance+\varepsilon )}$$

Odds ratio [exp(β*8*)] and 95% confidence intervals (CI) were reported, which are the change in the likelihood of tinnitus based on an increase in cognitive performance (per 1 z-score). The model was replicated using individual cognitive tests z-scores.

Sixth, to estimate the likelihood of poor cognitive performance based on tinnitus status, multivariable binary logistic regression models were used in the normal hearing and hearing loss sub-cohorts. The 25th percentile z-score of the normal hearing and no tinnitus group was calculated (i.e., z-score = −0.47) and used as a criterion to demarcate the outcome measure “poor cognitive performance,” in accordance with the recommended criterion for the definition of mild cognitive impairment (American Psychiatric Association, 1980):


Probability(Cognitivez-score≤-0.47)

$$=\frac{1}{1+e^{-(\beta 0+\beta 1*Age+\beta 2*Sex+\beta 3*Education+\beta 4*\mathrm{Physical}\mathrm{Well}-\mathrm{being}+}}$$

$${}^{\beta 5*\mathrm{PTA}+\beta 6*\mathrm{Race}+\beta 7*\mathrm{Hearing}\mathrm{Aid}+\beta 8*\mathrm{Tinnitus}+\varepsilon )}$$

Odds ratio [exp(β*8*)] and 95% confidence intervals (CI) were reported, which are the odds of poor cognitive performance of participants with no tinnitus relative to that of participants with tinnitus. The model was replicated for individual cognitive test z-scores. Note again that race was used only in the NHANES cohort, and hearing aid was used only in the hearing loss sub-cohorts.

**FIGURE 5:**
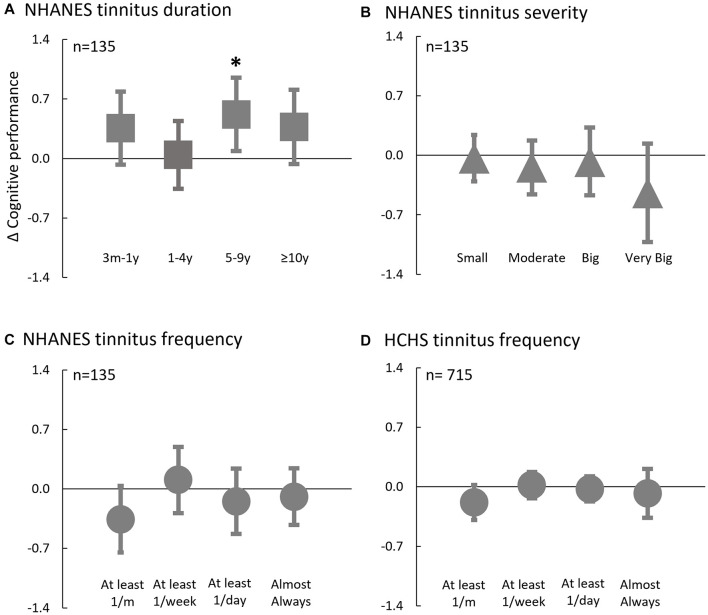
Adjusted Difference in Cognitive Performance Associated with Tinnitus Attributes in NHANES **(A–C)** and HCHS **(D)**. Multivariable linear regression models were used in participants with tinnitus to predict the relative difference in cognitive performance to the reference groups based on tinnitus attributes. Reference groups: **(A)** Tinnitus Duration < 3 m; **(B)** Tinnitus Severity = No problem; **(C,D)** Tinnitus Frequency < 1/month; Error bars denote 95% CI. *indicates a significant association between tinnitus factor and cognition and the tinnitus-based category scoring statistically significantly different than the reference group (*p* < 0.05).


Lastly, for those with tinnitus, sensitivity analysis was performed on the relationship between tinnitus-specific attributes and change in cognitive performance using multivariable linear regression models (Figure 5):

Cognitive⁢performance⁢(z-score)=β⁢0+β⁢1*Age+

β⁢2*Sex+β⁢3*Education+β⁢4*Physical⁢Well-being+

⁢β⁢5*PTA+β⁢6*Race+β⁢7*Tinnitus⁢attribute+ε

For the NHANES cohort (*n* = 135), tinnitus attributes included tinnitus duration, severity, and frequency, whereas, for the HCHS cohort (*n* = 715), tinnitus attributes were confined to tinnitus frequency only. Regression β*7* coefficients and 95% confidence intervals (CI) were reported, which are the adjusted difference in z-score based on the specified tinnitus attribute relative to the reference category. Note that race was used only in the NHANES cohort.

Data were analyzed using IBM SPSS software package version 26.0 (IBM_Corp, 2019). Significance was defined at the *p* < 0.05 level.

## Results

### Demographics and Descriptive Test Scores

Table 2 (NHANES) and Table 3 (HCHS) show the demographic characteristics and test scores of the four groups stratified based on the hearing (normal hearing vs. hearing loss) and tinnitus (tinnitus vs. not tinnitus) status. In each of the NHANES and HCHS cohorts, there was little or no difference between the four stratified groups in the covariates (age, education, physical well-being score), except for gender. In the NHANES cohort, there were more females in the hearing loss group vs. more males in the normal hearing group. The HCHS cohort showed an opposite pattern.

The mean cognitive performance in the NHANES cohort was zero for the normal hearing and no tinnitus group, as well as the hearing loss and tinnitus group, 0.1 for the normal hearing and tinnitus group, but −0.3 for the hearing loss and no tinnitus group (bottom row in Table 2). In the HCHS cohort, regardless of tinnitus, the mean cognitive performance was 0.1 for the normal hearing group and −0.1 to −0.2 for the hearing loss group (bottom row in Table 3).

These trends in the mean performance can also be seen from the raw cumulative frequency distribution curves in Figures 2A,B. The x-axis value corresponding to the 50% cumulative frequency (dashed line) represents roughly the mean performance.

### Tinnitus Correlates With Improved Cognition in Non-Hispanic Elderly With Hearing Loss

Figure 2C shows that while no association between tinnitus and cognition was observed in the normal hearing sub-cohort (β, 0.1; 95% CI, −0.1–0.2; *p*, 0.42), the hearing loss sub-cohort produced an unexpected result, namely, tinnitus was associated with better cognitive performance compared to no tinnitus (thick solid red line vs. dashed thin red line: β, 0.3; 95% CI, 0.05–0.5; *p*, 0.017). At the individual test level, only the CERAD word-learning test produced a significant association between tinnitus and improved cognitive performance for the hearing loss sub-cohort (β, 0.4; 95% CI, 0.02–0.7; *p*, 0.04). All the other individual tests showed no statistically significant association between tinnitus and cognition in the two sub-cohorts.

Figure 2D shows that within the HCHS cohort, tinnitus (dashed vs. solid line) was not associated with cognitive performance in the normal hearing (β, 0.0; 95% CI, 0.0–0.1; *p*, 0.42), nor the hearing loss (β, 0.0; 95% CI, −0.1–0.1; *p*, 0.83) sub-cohorts. The lack of a tinnitus effect on cognition in the HCHS cohort begs a question: Would the same result be obtained for the Hispanic sub-cohort in the NHANES database?

Figures 2E,F show the cumulative frequency distribution in the NHANES non-Hispanic (*n* = 495) and Hispanic (*n* = 148) sub-cohorts, respectively. In the non-Hispanic hearing loss sub-cohort (*n* = 109), tinnitus (*n* = 41) was significantly associated with better cognitive performance (β, 0.3; 95% CI, 0.1–0.5; *p*, 0.018), but this significant tinnitus effect disappeared, as expected from the HCHS result, in the NHANES Hispanic hearing loss sub-cohort (*n* = 26; β, 0.1; 95% CI, −0.5–0.8; *p*, 0.62). Normal hearing groups, Hispanic (*n* = 122) and non-Hispanic (*n* = 386), showed no association between tinnitus and cognition.

Figure 3 shows the adjusted cognitive performance of the four test groups. There was a significant main group effect on cognitive performance in both NHANES [*F*(3, 642), 11.8; *p* < 0.001, η2 = 0.1], and HCHS [*F*(3, 1714), 46.4; *p* < 0.001; η2 = 0.1]. *Post hoc* tests showed that for NHANES (Figure 3A), the hearing loss group and no tinnitus was the only group to score statistically significantly lower than the normal hearing groups (*p* < 0.001) and the group of hearing loss and tinnitus (*p*, 0.016). For the HCHS cohort (Figure 3B), both the hearing loss groups, with or without tinnitus, scored lower than the normal hearing groups (*p* < 0.001).

### Tinnitus Decreases the Odds of Poor Cognitive Performance

Figure 4 shows the adjusted individual probability of tinnitus as a function of cognitive performance in the NHANES normal hearing (Figure 4A) and hearing loss (Figure 4B), and the HCHS normal hearing (Figure 4C) and hearing loss (Figure 4D) sub-cohorts. Three of the four sub-cohorts (Figures 4A,C,D) had an odds ratio close to 1 (*p* > 0.05), indicating no significant association between tinnitus and cognition. The exception was the NHANES hearing loss sub-cohort (Figure 4B), in which each unit increase in the z-score increased a participant’s odds of having tinnitus by 2.3 times (OR, 2.3; 95% CI, 1.2–4.5; *p*, 0.014). At the individual test level, only the CERAD word-learning test showed a significant association, in which each unit increase in the z-score increased tinnitus odds by 1.6 times (OR, 1.6; 95% CI, 1.0–2.5; *p*, 0.03).

Using the 25th percentile threshold for poor cognitive performance, tinnitus status was not associated with poor cognitive performance in the HCHS normal hearing (OR, 0.9, 95% CI, 0.7–1.2; *p*, 0.56), hearing loss sub-cohorts (OR, 1.0, 95% CI, 0.7–1.2; *p*, 0.96), and the NHANES normal hearing sub-cohort (OR, 1.4; 95% CI, 0.7–2.6; *p*, 0.33). However, tinnitus status was a significant predictor in the NHANES hearing loss sub-cohort, in which a participant with tinnitus is 5.6 times less likely to have poor cognitive performance than a participant without tinnitus (OR, 5.6; 95% CI, 1.9–17.2; *p*, 0.002).

### Tinnitus Duration Correlates With Cognitive Performance

Figure 5 shows the adjusted cognitive performance as a function of the tinnitus attributes in NHANES and HCHS. In the NHANES cohort, only tinnitus duration (Figure 5A) was associated with improved cognitive performance [*F*(4,140), 2.6; *p*, 0.037]; relative to a participant with tinnitus duration less than 3 months, a participant with tinnitus duration of 5–9 years had better cognitive z-score by 0.5 (95% CI, 0.1–1.0; *p*, 0.02). Cognitive performance was not significantly associated with tinnitus severity [*F*(4,136), 0.9; *p*, 0.47; Figure 5B] or frequency [*F*(4,132), 1.5; *p*, 0.22; Figure 5C] in the NHANES, or with tinnitus frequency in the HCHS cohort [*F*(4,703), 1.2; *p*, 0.31; Figure 5D].

## Discussion

Against the main hypothesis, we found no evidence that tinnitus is associated with poor cognition, and if anything, tinnitus is associated with improved cognitive performance in the NHANES (non-Hispanic) elderly population with hearing loss (Figures 2C,E, 4B). Relative to participants without tinnitus in this population, tinnitus decreased the odds of having poor cognitive performance by 5.6 times. Tinnitus association with improved cognition was mainly with episodic memory (CERAD-word learning test), which is the main domain affected in mild cognitive impairment and Alzheimer’s disease dementia (Albert et al., 2011). Sensitivity analysis showed that, among those with tinnitus, cognitive performance was improved with longer tinnitus duration.

### Tinnitus Association With Better Cognition in the Elderly With Hearing Loss

The mechanisms responsible for hearing loss-associated cognitive decline are not well understood. This lack of understanding makes it difficult to delineate the mechanisms underlying tinnitus association with better cognition in age-related hearing loss (Whitson et al., 2018; Harris et al., 2019; Griffiths et al., 2020).

One possibility is tinnitus is a side effect of compensating for cortical changes in response to hearing loss that counteracts the increased central activities, and cortical tonotopic reorganization in response to hearing loss (Robertson and Irvine, 1989; Eggermont and Roberts, 2004). Koops et al. (2020a, b) showed that participants with hearing loss but no tinnitus, unlike those with tinnitus, had significantly different tonotopic maps, smaller gray matter volume, and thinner cortical surface both within and outside of the auditory pathway than controls. The presently observed association between longer tinnitus duration and better cognitive performance is consistent with the compensatory role of tinnitus in hearing loss-induced cortical plasticity. Another possibility is that tinnitus compensates for reduced auditory input due to hearing loss (Noreña, 2011; Zeng, 2020), which may, in turn, prevent auditory activity-related cognitive decline, for example, mnemonic memory (Gray et al., 2020; Veríssimo et al., 2021). This possibility is in line with the present finding that tinnitus improves episodic memory in individuals with hearing loss. A third possibility is that tinnitus is associated with less speech perception difficulties in those with hearing loss. While there is evidence for normal speech performance in individuals with tinnitus (Zeng et al., 2020), it is not known whether hearing loss individuals without tinnitus perform poorly in speech perception. If so speech perception difficulties may contribute to social disengagement that results in an accelerated cognitive decline (Bernabei et al., 2014). This third possibility would predict a global relationship between tinnitus and cognition.

### Race Effect on Hearing Loss and Tinnitus

It is difficult to explain the race dependency of tinnitus compensation for hearing loss-associated cognitive decline. At present, few studies examined the race or ethnicity variations in hearing loss and tinnitus, producing inconsistent findings, especially related to the Hispanic community. For example, one report found that hearing loss is less common among Mexican Americans than non-Hispanic whites (Davanipour et al., 2000), another showed similar prevalence between the Hispanic and other populations (Cruickshanks et al., 2015). The present study showed a similar discrepancy that 21.0% of the NHANES and 26.4% of the HCHS participants had hearing loss, but 23.8% of the NHANES participants reported tinnitus, compared to 43.2% of the HCHS participants. Other studies have also reported that non-Hispanic whites have higher odds of frequent tinnitus compared with other racial/ethnic groups (Shargorodsky et al., 2010). Golub et al. (2020) found after adjusting for covariates there was no statistically significant association between PTA and cognition in the NHANES but a significant association in the HCHS. The inconsistent finding between the two databases can be partially accounted for by the present result that the hearing-impaired non-Hispanic population with tinnitus had better cognitive performance than that without tinnitus, which would reduce the overall effect of hearing loss on cognition in NHANES.

### Limitations and Future Directions

There are two major limitations. First, this is a retrospective cross-sectional study with a narrow age range (60–69 years) and a relatively limited sample, where only an association rather than a causal relationship between tinnitus and cognition could be determined. Second, tinnitus characterization is based on a simple binary question on the absence or presence of tinnitus. Longitudinal data are needed to directly address whether and how tinnitus is associated with hearing loss-associated cognitive decline, and their interactions with age, sex, and race factors. Brain imaging and electrophysiological studies will likely shed light on mechanisms underlying both hearing loss and tinnitus, as well as their functional and structural relationship to dementia (Slade et al., 2020). Future studies need to investigate if tinnitus simulations in participants with hearing loss but no tinnitus might delay or even prevent dementia. Possibly, both actual and simulated tinnitus may counteract the neuroplastic changes in response to auditory deprivation (Guitton, 2012). Additionally, future studies should include rehabilitation outcome measures that go beyond hearing and tinnitus assessment to include cognitive domains, particularly in the elderly (Naples et al., 2021). Finally, because the impact of hearing loss on cognition might be higher without tinnitus than with tinnitus, clinicians should pay special attention to individuals with hearing loss but no tinnitus to reduce the risk of cognitive decline.

### Conclusion

The present study found that not only does tinnitus not aggregate hearing loss-related cognitive decline, but rather it is associated with better cognitive performance than those with hearing loss and no tinnitus, at least in the non-Hispanic elderly population. The present finding challenges the present assumption that tinnitus impairs cognitive function and provides interesting directions for future studies.

## Data Availability Statement

Publicly available datasets were analyzed in this study. This data can be found here: NHANES Database from https://www.cdc.gov/nchs/nhanes/index.htm and HCHS-SOL Database from https://biolincc.nhlbi.nih.gov/home/.

## Ethics Statement

Ethical review and approval was not required for the study on human participants in accordance with the local legislation and institutional requirements. Written informed consent for participation was not required for this study in accordance with the national legislation and the institutional requirements.

## Author Contributions

YH and F-GZ contributed to the concept and design, acquisition, analysis, and interpretation of data. YH contributed to the statistical analysis and drafted the manuscript. F-GZ supervised and critically revised the manuscript and obtained the funding. Both authors contributed to the article and approved the submitted version.

## Conflict of Interest

F-GZ owns stock in Axonics, DiaNavi, Nurotron, Syntiant, Velox, and Xense. The remaining author declares that the research was conducted in the absence of any commercial or financial relationships that could be construed as a potential conflict of interest.

## Publisher’s Note

All claims expressed in this article are solely those of the authors and do not necessarily represent those of their affiliated organizations, or those of the publisher, the editors and the reviewers. Any product that may be evaluated in this article, or claim that may be made by its manufacturer, is not guaranteed or endorsed by the publisher.
